# Expression of Concern: Jiang, H.; Mei, Y.-F. SARS-CoV-2 Spike Impairs DNA Damage Repair and Inhibits V(D)J Recombination In Vitro. *Viruses* 2021, *13*, 2056

**DOI:** 10.3390/v14010012

**Published:** 2021-12-22

**Authors:** Eric O. Freed, Oliver Schildgen

**Affiliations:** 1 HIV Dynamics and Replication Program, Center for Cancer Research, National Cancer Institute, Frederick, MD 21702-1201, USA; 2 Institut für Pathologie, Kliniken der Stadt Köln gGmbH, Klinikum der Privaten Universität Witten/Herdecke mit Sitz in Köln, Ostmerheimer Str. 200, D-51109 Cologne, Germany

We are issuing this expression of concern in consultation with the publisher to fulfil their reporting obligation regarding the publication [1] mentioned above. 

One of the authors has raised concerns regarding the methodology employed in the study, the conclusions drawn and the insufficient consideration of laboratory staff and resources. 

In order to keep the highest scientific standards, an in-depth investigation is initiated by the responsible editors together with the journal’s editorial office in collaboration with the editorial board, and in accordance with the Committee on Publication Ethics (COPE) guidance. The article will be updated and any necessary corrections made at the conclusion of the investigation process.
